# Biofeedback for Post-stroke Gait Retraining: A Review of Current Evidence and Future Research Directions in the Context of Emerging Technologies

**DOI:** 10.3389/fneur.2021.637199

**Published:** 2021-03-30

**Authors:** Jacob Spencer, Steven L. Wolf, Trisha M. Kesar

**Affiliations:** ^1^Division of Physical Therapy, Department of Rehabilitation Medicine, Emory University School of Medicine, Atlanta, GA, United States; ^2^Department of Medicine, Emory University School of Medicine, Atlanta, GA, United States; ^3^Department of Cell Biology, Emory University School of Medicine, Atlanta, GA, United States; ^4^Center for Visual and Neurocognitive Rehabilitation, Atlanta VA Health Care System, Decatur, GA, United States

**Keywords:** gait rehabilitation, real-time biofeedback, locomotion, hemiparesis, cerebrovascular accident

## Abstract

Real-time gait biofeedback is a promising rehabilitation strategy for improving biomechanical deficits in walking patterns of post-stroke individuals. Because wearable sensor technologies are creating avenues for novel applications of gait biofeedback, including use in tele-health, there is a need to evaluate the state of the current evidence regarding the effectiveness of biofeedback for post-stroke gait training. The objectives of this review are to: (1) evaluate the current state of biofeedback literature pertaining to post-stroke gait training; and (2) determine future research directions related to gait biofeedback in context of evolving technologies. Our overall goal was to determine whether gait biofeedback is effective at improving stroke gait deficits while also probing why and for whom gait biofeedback may be an efficacious treatment modality. Our literature review showed that the effects of gait biofeedback on post-stroke walking dysfunction are promising but are inconsistent in methodology and therefore results. We summarize sources of methodological heterogeneity in previous literature, such as inconsistencies in feedback target, feedback mode, dosage, practice structure, feedback structure, and patient characteristics. There is a need for larger-sample studies that directly compare different feedback parameters, employ more uniform experimental designs, and evaluate characteristics of potential responders. However, as these uncertainties in existing literature are resolved, the application of gait biofeedback has potential to extend neurorehabilitation clinicians' cues to individuals with post-stroke gait deficits during ambulation in clinical, home, and community settings, thereby increasing the quantity and quality of skilled repetitions during task-oriented stepping training. In addition to identifying gaps in previous research, we posit that future research directions should comprise an amalgam of mechanism-focused and clinical research studies, to develop evidence-informed decision-making guidelines for gait biofeedback strategies that are tailored to individual-specific gait and sensorimotor impairments. Wearable sensor technologies have the potential to transform gait biofeedback and provide greater access and wider array of options for clinicians while lowering rehabilitation costs. Novel sensing technologies will be particularly valuable for telehealth and home-based stepping exercise programs. In summary, gait biofeedback is a promising intervention strategy that can enhance efficacy of post-stroke gait rehabilitation in both clinical and tele-rehabilitation settings and warrants more in-depth research.

## Introduction

**Figure 1:**
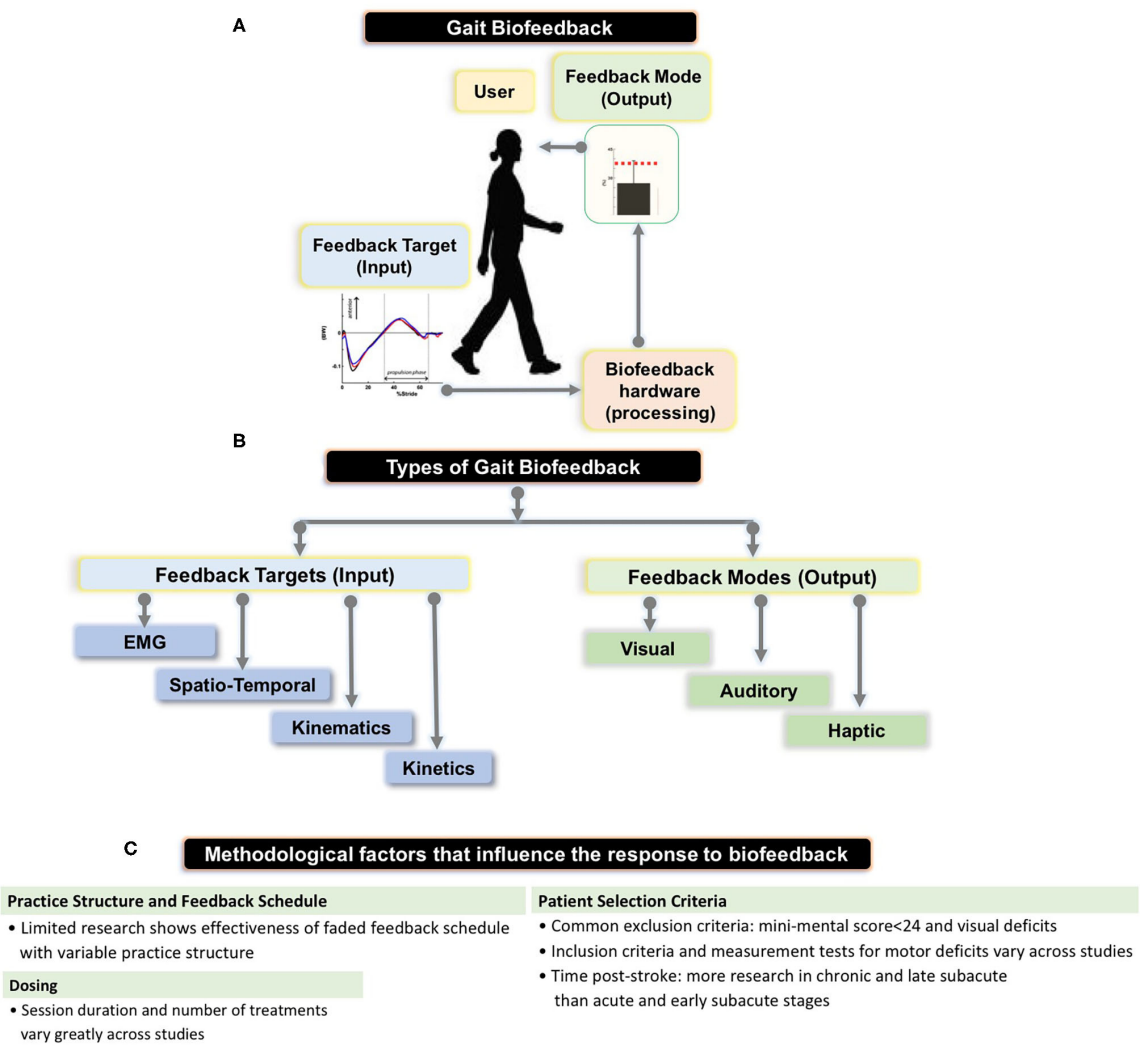
**(A)** Schematic showing the setup for gait biofeedback, where a targeted gait parameter (e.g., anterior-posterior ground reaction force or electromyographic (EMG) activation) is measured, processed, and real-time, accurate information about the ongoing gait parameter is provided to the user *via* a feedback mode (e.g., audio-visual interface). **(B)** The flowchart shows types of feedback targets—EMG, spatio-temporal (e.g., step length, cadence), kinematic (e.g., joint angles), kinetic (e.g., ankle moment) as well as different feedback modes (e.g., visual, audio, haptic) that we summarize in our review, and provided by Stanton et al. **(C)** Summary of methodological parameters that we identified as factors influencing previous research results on biofeedback targeting stroke gait deficits.

Biofeedback is a process by which an external stimulus derived from previously covert physiological or motor performance data is provided to an individual in real time to induce self-modification of a behavior (1–6). Applications of biofeedback can be as disparate as cuing an individual with a tension headache to relax offending muscles through electromyographic (EMG) feedback and retraining standing balance through visual feedback regarding ongoing foot center of pressure data (3, 7). Biofeedback can also be used in locomotor rehabilitation to promote restitution of appropriate gait patterns (1). During gait biofeedback training, a target variable such as push off force or knee angle at mid-stance, is conveyed as a comprehensible visual, auditory, or tactile signal alerting the user of their relative success in reaching the targeted gait parameter (4, 8) (Figure 1A). Accurate and instantaneous biofeedback can be provided for every step with the goal of addressing deleterious gait abnormalities, which often require frequent and specific cuing to correct (1, 9–11).

Stroke is a leading cause of adult disability and a majority of stroke survivors present with post-stroke gait dysfunction resulting in compromised community participation and quality of life (2, 12–14). The prevalence and impact of residual gait deficits after stroke positions post-stroke individuals as ideal candidates for gait biofeedback as a treatment modality, especially as an adjunct to other clinically-relevant and evidence-supported gait rehabilitation treatments such as high intensity locomotor training (15, 16). Despite the compelling rationale for biofeedback as a gait training tool, its use is relatively uncommon in clinical practice.

While there is little research on barriers limiting clinical use of biofeedback, costly and cumbersome equipment combined with a lack of research consensus may be contributing factors. However, technological advances, including the evolution of wearable sensors, may resolve the former problem and serve to make biofeedback gait training a more realistic intervention choice for clinicians (17). An additional benefit of wearable sensor-based biofeedback may include the ability to convey clinician-selected cues during a home exercise or telehealth program, enhancing the efficacy of clinical rehabilitation and carryover of therapeutic gains to community ambulation (17). Of course, the newfound accessibility granted by wearable sensor technology and telehealth applications will be of little value if biofeedback itself is an ineffective post-stroke gait training intervention.

Therefore, the objectives of this review are 2-fold: (1) to evaluate the current state of biofeedback literature that pertains to post-stroke gait training; and (2) to determine future research directions related to gait biofeedback with a special focus on application of new technologies. To achieve these objectives, the literature search was designed to include multiple feedback modes, target parameters for gait retraining, and methods of post-stroke gait biofeedback as possible, with the intent to synthesize our current knowledge regarding the methodological parameters and effects of post-stroke gait biofeedback, while also outlining areas for future research.

## Literature Search Methods

This literature review categorizes the factors that influence the efficacy of biofeedback as a gait training intervention for post-stroke individuals and identifies knowledge gaps in the evidence landscape. The literature review included searches of databases, including PubMed and PEDro. All articles available on PEDro were categorized based on their PEDro score. The following search terms were used in PubMed: (biofeedback[tw] OR “Biofeedback, Psychology”[Mesh] OR “electromyographic biofeedback”[tw] OR “EMG BFB”[tw] OR EMGBFB[tw] OR EMG-BFB[tw]) AND (“stroke rehabilitation”[tw] OR “Stroke Rehabilitation” [Mesh] OR “Stroke/rehabilitation” [Mesh]) AND (gait[tw] OR “Gait”[Mesh] OR walking[tw] OR “Walking”[Mesh] OR “mobility limitation”[tw] OR “Mobility Limitation”[Mesh]) AND English[lang] AND (“1970/1/1”[Date - Publication]: “2020/4/1”[Date - Publication]) NOT (“animals”[MeSH Terms] NOT “humans”[MeSH Terms]).

### Eligible Studies

All articles that appeared in the initial database search were evaluated for relevance by author JS. Randomized Control Trials (RCTs) scoring >6/10 on the PEDro scale were given preference. Because our goal was to capture a wide variety of experiment designs lower scoring RCTs and non-RCTs were also included if they used unique or rigorous methodology. Articles exclusively involving biofeedback provided during static standing or marching in place as well as provided in conjunction with robotic gait training were excluded.

We were unable to find systematic reviews specifically addressing biofeedback for post-stroke gait rehabilitation, although two reviews covered somewhat related topics and merit mention. Woodford and Price (14) performed a review evaluating the efficacy of EMG biofeedback training following stroke, and found that overall, biofeedback did not demonstrate treatment benefits relative to standard physical therapy. However, the Woodford and Price review was not specific to gait and included studies assessing the efficacy of EMG biofeedback in improving upper limb activity (14). Woodford and Price also focused exclusively on EMG biofeedback (14). Another systematic review by Stanton et al. (2) found that lower extremity biofeedback delivered during movement from sitting to standing, standing, or ambulation improved lower limb activity in individuals post-stroke compared to traditional therapy, but in that review, nine of the 18 trials cited were unrelated to ambulation (2) and only four studies assessed progress at any time point other than immediately following the intervention (2). As a result, Stanton et al. failed to find support for the long-term efficacy of lower limb biofeedback (2). Finally, a mapping review by van Gelder et al. (18), sought to categorize existing gait biofeedback literature by both quality and content, but was not exclusive to individuals post-stroke (18). While this review is valuable, post-stroke individuals present unique sensorimotor and gait deficits (19), which merit separate analysis and synthesis, and was the specific focus of the current manuscript.

While there have been a few literature reviews on biofeedback as a rehabilitation modality, previous work has not focused exclusively on stroke as a neuropathology and biofeedback as a basis for specifically targeting gait deficits. Because a goal of the current review was to capture a wide variety of potential methods for providing biofeedback during post-stroke gait retraining, our inclusion criteria that relate to quality and study design are broader, including both RCTs and non-RCTs, with the intent of evaluating a greater number of studies Tables 1 and 2. Our overall goal was to determine whether gait biofeedback is effective at improving stroke gait deficits while also probing why and for whom gait biofeedback may be an efficacious treatment modality. We first identify, categorize, and summarize factors impacting the results of previous research related to post-stroke gait biofeedback.

**Table 1:** Summary of relevant characteristics of selected randomized control trials included in the review.

**Study**	**Feedback target**	**Feedback mode**	**Motor learning strategies**	**Dosing**	**Time post-stroke**	**Key participant inclusion and exclusion criteria**	**Control group**	**Sample size**		**Key outcomes and descriptive statistics**		**Statistical results**
								**Feedback Group**	**Control Group**	**Experimental Group**	**Control Group**	
										10 meter walk test (seconds) (Pre to Post)		
Choi et al. (20)	Kinetic (weight-bearing during stance)	Auditory	Walking only allowed to continue when 50% of total body weight was placed through stance phase leg	18 sessions, 20 min/session for 3 weeks	Not specified, patients recruited from rehabilitation center	*Inclusion:* Brunnstrom score between 3 and 5, able to walk independently *Exclusion:* Modified ashworth >1+ in dorsiflexor, Mini-Mental score <24	General overground gait training	*n* = 12	*n* = 12	23 (14.6) to 17.2 (10.1)	18.1 (16.4) to 16.9 (15.6)	Significant improvement in 10 min walk test (*p* = 0.02), functional gait assessment, COP path length with eyes open and eyes closed in experimental group relative to control
										Change in step symmetry index (Pre-post)		
Druzbicki et al. (21)	Spatio-temporal (Step length)	Visual	Step-length and gait speed increased, bodyweight support decreased progressively based on performance. Feedback present throughout training.	15 sessions, 30 min/session, for 3 weeks	Subacute	*Inclusion*: Brunnstrom 2-3, able to walk unassisted *Exclusion*: Visual deficits, Mini-Mental score <24	Body-weight supported treadmill training without biofeedback	*n* = 15	*n* = 15	0.03 (0.02)	0.02 (0.02)	No significant difference (*p* = 0.902) in step-symmetry index between groups
										Step-symmetry index (at Pre and 6-month follow up)		
Druzbicki et al. (22)	Spatio Temporal (Step-length)	Visual	Constant feedback, speed and step length adjusted according to task performance	10 sessions, 20 min/session, for 2 weeks	Chronic	*Inclusion:* Independent gait, Brunnstrom 3-4 *Exclusion:* Visual disturbances, Mini-Mental score <24	Treadmill training	*n* = 15	*n* = 15	1.5 (0.36) to 1.26 (0.12)	1.36 (0.2) to 1.35 (0.28)	No significant changes in step-symetry index between experimental and control group
										Gait velocity (%h/s): Pre to 6-month follow up		
Jonsdottir et al. (23)	EMG (Plantar Flexors)	Auditory	Variable practice and faded feedback	20 sessions, including ≥15 min of gait training	Chronic	*Inclusion:* Able to walk 10 m without assistance, volitional triceps surae contraction *Exclusion:* Visual or auditory deficits, Mini-mental score <24	Standard care	*n* = 10 (at Post) *n* = 9 (at 6-month follow-up)	*n* = 10 (at Post) *n* = 9 (at 6-month follow-up)	28.7 (10.8) to 38.8 (8.9)	26.3 (11.9) to 28.4 (14.3)	Significant (*p* < 0.05) increase in gait velocity, step length, and peak ankle power in treatment group. No such changes in control group
										Reduction in hyperextension (degrees)		
Morris et al. (24)	Kinematic (Knee angle)	Auditory	Therapy and control group treatments based on principles of Motor Relearning programme (MRP)	45 min of therapy, 5 days per week, for 4 weeks, with >30 min spent on knee control	Subacute	*Inclusion:* Able to ambulate 10 m without assistance, knee hyperextension warranting treatment *Exclusion:* Auditory deficits	Physical therapy based on MRP principles	*n* = 13	*n* = 13	1.7 (+/- 1.8)	0.4(+/- 3.1)	Treatment group showed signficant (*p* = 0.011) reduction in knee hyperextension relative to the control group after phase 2
										Step symmetry ratio pre to post intervention		
Brasileiro et al. (25)	Spatiotemporal (foot placement, metronome)	Auditory, Visual, Control (three groups)		20 min	Chronic	*Inclusion:* Gait levels 4-5 on Functional Ambulatory Category, slow to moderate gait speed (<0.8 m/s), Mini-Mental score>23–24/30 (dependent on social history) *Exclusion:* Visual/auditory deficits	Partial bodyweight supported treadmill training	*n* = 10 (Group 1) *n* = 20 (Group 2)	*n* = 10	Group 1: 1.43 (0.25) to 1.34 (0.23) Group 2: 1.49 (0.34) to 1.58 (0.47)	1.61 (0.43) to 1.53 (0.41)	Treatment group displayed no significant change in stride length relative to the control group
										Change in gait speed (cm/s)		
Sungkarat et al. (26)	Ambulation (Non-paretic leg swing phase duration in gait, paretic leg weight bearing in standing)	Auditory	Sensors adjusted to progressively challenge participant	15 sessions, 30 min of gait training in each	equalized <6 months and >6 months post-stroke	*Inclusion:* Orpington prognostic score 3.2-5.2 *Exclusion*: Impaired cognition/communication	Conventional gait training	*n* = 17	*n* = 18	12.24 (11.7)	4.06 (6.0)	Treatment group displayed significant (*p* < 0.05) improvements in standing symetry, gait symmetry, and gait speed compared to the control group
										Change in gait speed pre to post intervention (cm/s)		
Jung et al. (27)	Kinetic (force on pressure sensing cane)	Auditory	Feedback threshold derived from objective data, and modified weekly if patient exhibited <20% error rate	30 min/session, 5 days per week, for 4 weeks	Not explicitly specified, but patients recruited from rehabilitation center	*Inclusion:* Functional classification 2-3 *Exclusion: V*estibular symptoms, Mini-Mental score >24	Gait training	*n* = 11	*n* = 10	13.5 (7.1–19.9)	3.7 (2.3–9.7)	Treatment group displayed significant improvements (*p* < 0.05) in peak force of cane, EMG activity in stance leg, single limb support phase and gait speed

*The table lists that received scores of ≥6/10 (moderate to high quality) on the Physical Therapy Evidence Database (PEDro) scale. All studies included in this table evaluated the relative efficacy of a biofeedback intervention provided during non-robotic gait training and compared to a non-biofeedback control group. The descriptive statistics are listed as average (standard deviation)*.

**Table 2:** Summary of relevant characteristics of other selected studies.

**Study**	**Feedback target**	**Feedback mode**	**Time post-stroke**	**Control group**	**Key inclusion and exclusion criteria**	**Results**
Afzal et al. (28)	Kinetic	Combined Haptic	Subacute	Ambulation without biofeedback, Repeated measures design	Able to ambulate 10 ft without assistance, Brunnstrom stage >3	Kinesthetic cues induced significant improvements in paretic muscle activity and mediolateral trunk control during walking.
Aruin et al. (29)	Spatiotemporal	Auditory	Subacute	Ambulation training without biofeedback	Able to ambulate 4.5–6 m without assistance and follow verbal instructions	Biofeedback group had significantly greater step-width following treatment.
Bradley et al. (30)	EMG	Visual or Auditory	Subacute	Same treatment techniques without biofeedback	No global amnesia or dementia	No significant difference between groups in mobility or activities of daily living.
Genthe et al. (1)	Kinetic	Visual and Auditory	Chronic	Ambulation without biofeedback. Repeated measures design	Able to ambulate continuously on treadmill for 6-min, able to communicate with investigators	Significant improvement in peak AGRF with biofeedback condition.
Ma et al. (8)	Kinetic	Haptic	Chronic	Ambulation with Biofeedback turned off. Repeated measures design	Able to ambulate 10 m independently	Significant reduction in foot inversion with biofeedback condition.
Wolf and Binder-MacLeod (31)	EMG	Visual and Auditory	Chronic	Three other groups: No biofeedback, general relaxation, upper extremity biofeedback	No previous exposure to EMG biofeedback	Experimental group did not experience significant increase in walking speed, but did reduce dependence on assistive devices.

*The table lists non-RCTs, RCTs that scored <6/10 on the PEDro scale, and RCTs that were not scored by PEDro. Similar to Table 1, all studies included in this table evaluated the relative efficacy of a biofeedback intervention provided during non-robotic gait training compared to a non-biofeedback control group or condition. The descriptive statistics are listed as average (standard deviation)*.

## Types of Gait Biofeedback

### Target Parameters During Gait Biofeedback

Previously, Tate and Milner (2) identified the four most prevalent biofeedback target parameters for gait training: (1) EMG biofeedback, which targets magnitude of activation in a specific muscle; (2) kinematic biofeedback, which targets segment motions or joint angles during specific phases of the gait cycle (e.g., hip, knee, or ankle angles during swing phase); (3) kinetic biofeedback, which targets forces generated during a phase of gait (e.g., push-off force or downward pressure exerted on an assistive device during the stance phase of gait); and (4) spatiotemporal biofeedback, which relates to the spatial and temporal aspects of gait (e.g., step length, cadence) (Figure 1A). Of the 4 forms of gait biofeedback, EMG biofeedback may be the most well-researched in post-stroke individuals, with individual RCTs demonstrating mixed results (14, 23, 30–32). One possible explanation for the unremarkable results from EMG biofeedback studies may be the focality of the treatment target. Because EMG biofeedback only targets one muscle group at a time (usually the triceps surae), this treatment may provide the greatest benefit to individuals whose gait deficits are largely impacted by functional weakness in a single muscle group and have adequate residual strength to perform a volitional contraction. This approach often neglects the importance of retraining other hemiparetic muscle groups, such as the quadriceps or hamstrings. As a result, participant inclusion and exclusion criteria, which are not uniform across these studies, likely have a significant influence on treatment success (33). Another possible explanation for variable effects of EMG biofeedback lies in the fact that while having been in existence for decades, the technologies and protocols underlying EMG biofeedback may have evolved and contributed additional variability in study methods and research results (14, 23, 31, 34).

There are fewer studies from which conclusions can be drawn using other forms of gait biofeedback. Morris et al. (24) and Basaglia et al. (35) both found that electrogoniometric biofeedback (a form of kinematic biofeedback) significantly reduced genu recurvatum in post-stroke individuals, but little additional research has been done on the topic. Spatiotemporal biofeedback that targets paretic step length or step width has yielded inconsistent results in people post-stroke (4, 21, 36). A recent high-quality randomized control trial performed by Druzbicki et al. (21) failed to demonstrate significant improvements in gait quality following step length biofeedback compared to conventional treadmill training. Importantly, Druzbicki et al.'s (21) study differed from many other biofeedback RCTs by involving patients in the sub-acute phase of recovery and combined biofeedback with body weight supported gait training. The same study team also performed another high-quality RCT using similar feedback parameters (visual feedback targeting paretic step length during treadmill training) on individuals with chronic stroke, and found no significant long-term increases in step length and step symmetry compared to a control group of post-stroke individuals performing treadmill training without biofeedback when initial values were compared to those gathered after 6-month (22, 36). Similarly, Brasileiro et al. (25) found no significant improvement in stride length in post stroke individuals with the addition of spatiotemporal biofeedback to partial bodyweight supported treadmill training program. Biofeedback targeting step-width is less prevalent as a gait training modality for individuals post-stroke (29).

Kinetic biofeedback most commonly involves the use of force sensors placed either in the shoe or embedded within the surface over which the individual is ambulating, such as the floor or a treadmill. Sungkarat et al. (26) and a pilot study by Choi et al. (20) both used forms of kinetic biofeedback to retrain increased paretic leg weight bearing following stroke, with the former finding a significant increase in gait symmetry compared to the non-biofeedback control, and the latter finding significant improvements in the 10-meter walk test, functional gait assessment, and center of pressure path length relative to the non-biofeedback control. Jung et al. (27) employed a unique version of kinetic biofeedback in the form of a pressure sensing cane which provided auditory cues designed to reduce assistive device dependence in post-stroke individuals. Following 4 weeks of training, participants in the experimental group experienced significant improvements in both peak force through assistive device and in gait speed compared to the control group (27). Anterior ground reaction force (AGRF) biofeedback is another form of kinetic feedback which encourages greater push-off from the paretic leg (37), which in turn enables effective transition from the stance to swing phase of gait and faster gait speeds (1). Unlike EMG feedback targeting a single muscle (e.g., triceps surae), AGRF biofeedback targets force production from and orientation of the paretic limb as a whole, encouraging activation of multiple muscle groups involved in generating push off (1). Preliminary literature on AGRF gait biofeedback is promising, showing improvements in AGRF and propulsion-related variables only in the targeted (paretic) leg, albeit from a single-session small-sample study (1). There is also evidence that AGRF biofeedback can improve push off force in older adults who are not affected by stroke (38).

In summary, a review of previous biofeedback studies reveals that multiple target gait parameters have been utilized with the goal of improving post-stroke gait impairments with mixed results. Several additional methodological factors discussed in subsequent subsections can impact the efficacy of gait biofeedback.

### Modes of Feedback

During gait training, biofeedback output can be provided using auditory cues, a visual display interface, tactile stimuli, or a combination of multiple modes (Figure 1A). Auditory signals (1, 23) and visual displays (1, 21) are the two most commonly used means of conveying feedback to the patient, but other feedback modes have also been explored. Haptic biofeedback, presented as vibratory or tactile sensory stimuli delivered *via* surface electrodes, has been studied in conjunction with wearable plantar force sensors (8, 39). Additionally, virtual reality (VR) gait training, which constitutes an immersive 3-dimensional interface in which patients can perform real world activities (40), can be sub-categorized as a visual gait biofeedback mode if the VR involves integration of real-time gait performance data. A notable form of VR gait training is optic flow, which renders an artificial virtual environment which fails to match proprioceptive input in order to facilitate correction of gait abnormalities (41). Kang et al. (41) found that treadmill training with modulated optic flow resulted in significantly improved gait velocity compared to standard treadmill training. A recent systematic review concluded that VR-based training is more effective than balance or gait training without VR at improving balance and gait ability following stroke (40). However, the studies included in this review demonstrated considerable variability in both content and quality, likely because VR-based gait interventions are still in early developmental stages (40–43).

Only a few studies compare different modes of feedback in post-stroke individuals. A study (44) involving only nine post-stroke and seven able bodied individuals found no significant differences in AGRF in the post-stroke group during gait trials comprising visual, audiovisual, and auditory biofeedback but acknowledged that more systematic larger-sample investigations are needed to determine the most optimal feedback mode. Additionally, because visual and sensory deficits are common following stroke, clinicians must consider how each stroke survivor's deficits may interfere with processing a particular biofeedback mode (45, 46). For example, proprioceptive or visual field deficits may interfere with a stroke survivor's ability to capitalize on haptic or visual feedback cues, respectively. The training environment is another factor to consider when selecting the mode of feedback. For example, a user on a treadmill in a clinical setting may be an appropriate candidate for visual modes of biofeedback while a stroke survivor ambulating overground or unsupervised in the community may be a better candidate for auditory feedback delivered *via* a headset connected to their smart phone. Finally, the feedback mode may impact motor learning processes. Visual feedback may be more likely to create dependence on external cues than auditory feedback, as individuals who received concurrent visual feedback demonstrated worse performance on motor skill retention tests than those receiving auditory feedback (47, 48).

Another important factor in selecting a feedback mode is the coding scheme (i.e., the method by which the magnitude of error in ongoing task performance is processed and conveyed to the patient during biofeedback). In repeated measures study (*n* = 8), Afzal et al. (49) applied vibrotactile feedback to post-stroke individuals during ambulation training with the intention of correcting gait asymmetry. Feedback regarding the magnitude of ongoing error in inter-limb step length asymmetry was provided to study participants but using different mechanisms to code or transform the ongoing error in gait performance to vibrotactile feedback. Either the duration or frequency of vibrotactile feedback was modulated, using either positive or negative encoding. Thus, in addition to a bout of ambulation without feedback (control), each participant completed trials of overground ambulation while receiving biofeedback provided with each of the following vibrotactile coding schemes: (i) duration of vibration feedback increases proportionally with the magnitude of error, (ii) vibration duration is inversely related to error magnitude, (iii) vibration intensity increases proportionately with error magnitude, and (iv) vibration intensity is inversely related to error magnitude. Despite the small sample size, Afzal et al. found a significant effect of the type of vibratory feedback coding schemes on gait symmetry ratio. Interestingly, a significant improvement in gait symmetry ratio was observed during proportional feedback coding provided by varying vibration duration compared to vibration intensity (49), suggesting that, at least for vibrotactile or haptic modes of feedback, modulating the duration vs. the intensity of vibration duration coding may yield more favorable results. However, there is a need to evaluate these results in larger sample studies and using other forms of feedback (e.g., auditory). Studies comparing time-coded and intensity-coded biofeedback schemes to non-magnitude coded biofeedback that provides the same biofeedback signal type regardless of error size should also be undertaken. Feedback strategies of coding schemes, particularly duration-based coding of error magnitude may lead to superior motor learning compared to uniform error signals in patients who are able to process more complex signals as the former provides the patient with more information regarding aberrant movement patterns, but more confirmatory studies are needed.

The combination of a primary mode of feedback with a secondary feedback target intervention has been studied recently. Shin et al. (50) found that the addition of a rhythmic auditory cue to visual biofeedback led to a significant improvement in symmetry ratio compared to both visual biofeedback alone and non-biofeedback controls. Cherry-Allen et al. (51) simultaneously explicitly targeted knee joint angle *via* visual biofeedback and step length asymmetry implicitly *via* split belt walking, and found similar joint angle changes in response to both the biofeedback plus split-belt treadmill group and the biofeedback only group, indicating that individuals with stroke have the capacity to target two deficits concurrently in the same session. Afzal et al. (28) showed that combined use of a haptic cane device providing kinesthetic perception and a vibrotactile device providing tactile cues on the paretic leg increased gait speed and symmetry post-stroke. The combination system incorporated input from wireless EMG sensors and smartphone measurements of trunk sway to target sub-optimal gait patterns (28). These recent studies on relatively small, homogenous samples, suggest that more research is needed for an optimal individual-specific selection of multiple biofeedback targets or for combining biofeedback with a second intervention. There is also little research surrounding the effect of combining gait biofeedback with modalities, such as motor priming (52) or non-invasive neuromodulation techniques [e.g., transcranial direct current stimulation (53, 54), repetitive transcranial magnetic stimulation (55)], on the efficacy of gait biofeedback. Additionally, the feasibility of applying more complex biofeedback protocols in clinical settings requires considerable exploration before defining algorithms that can be followed successfully.

## Key Methodological Factors Influencing Response to Gait Biofeedback

### Practice Structure and Feedback Schedule During Gait Training

Careful manipulation of the structure of practice within a training session (e.g., how bouts of biofeedback gait training are organized within a session) and feedback schedule (how frequently biofeedback input is provided to the individual during and across training bouts) are important factors impacting the magnitude of locomotor learning (47); yet few previous efforts have incorporated strategies to enhance motor learning during the use of biofeedback for retraining stroke gait (4) (Figure 1B). Jonsdottir et al. (23) conducted an RCT that incorporated principles of motor learning during EMG biofeedback by using variable practice and a faded feedback schedule, and found significant increases in push off, gait velocity, and step length in the biofeedback group and no significant improvements in the control group, both immediately post-treatment and 6 weeks following treatment. However, because the gait training received by the control group consisted of strategies selected by clinicians at the rehabilitation center, the exact nature of which were not directly specified, the larger improvement in the experimental group could be due to variable practice gait training (changes in gait speed, direction, terrain and step length which may not have been a component of the control intervention) with the addition of biofeedback being potentially inconsequential (23). While there appears to be no RCT directly comparing practice structure or feedback schedules between two gait biofeedback training groups, Tsaih et al. (56) did compare variable and constant practice during biofeedback-based balance training and found that the group receiving variable force biofeedback to the tibialis anterior displayed better standing balance than the group receiving constant force biofeedback. While these studies alone cannot definitively prove that variable practice biofeedback gait training is superior to constant practice biofeedback gait training, their results underscore the need for further investigation (23, 56). Similarly, other potential permutations of practice parameters or feedback structure, such as massed and spaced practice schedules should be evaluated using one or more forms of biofeedback. Finally, the relative success of the Jonsdottir et al. study compared to other EMG biofeedback RCTs suggests that mode of feedback may be less important than other aspects of experiment design.

### Dosing

There is little research evaluating the effect of dosage on gait biofeedback training. In fact, “dose” is often an ill-defined a term in stroke rehabilitation studies (47). Here, dosing includes feedback duration (amount of time spent on biofeedback gait training during each session), training frequency (e.g., how often the biofeedback sessions occurred per week), and total number of treatment sessions (e.g., 3 vs. 18 training sessions). Many gait biofeedback studies for post stroke individuals only evaluated changes in gait variables in response to a single session of training, while multi-session studies displayed variability in the number of sessions and duration of practice per session (1, 23, 36). In general, multi-session studies comprised 10–20 sessions (1, 23, 24, 29, 30, 36), with each session being 11–30 min in duration. Treatment frequency varied from three times per week to twice per day (21, 29, 30, 37). There is insufficient evidence to comment on the relative efficacy of different dosing strategies or the optimal recommended dosage, a phenomenon not unique to biofeedback gait training. While some research findings indicates that increased therapy time may lead to improved outcomes in post-stroke individuals (47), the exact nature of the dose-response relationship for physical therapy and motor recovery following stroke is a broad area that requires further investigation (47). Dose response characteristics of physical therapy treatments, such as gait biofeedback post-stroke, may also be influenced by chronicity of stroke, and vary across the stages of recovery (47).

### Participant Selection Criteria

Bernhardt et al. outlined the chronological stages of stroke recovery from a rehabilitation perspective as follows: hyper acute (0–24 h), acute (1–7 days), early subacute (7 days to 3 months), late subacute (3–6 months), and chronic (>6 months) (57). The majority of high quality biofeedback research has been conducted on individuals in the chronic and late subacute stages of stroke rehabilitation (2, 4, 14). There is a scarcity of RCTs in the early recovery stages for post-stroke motor interventions (58), a phenomenon that remains true for biofeedback studies. A recent high-quality biofeedback study in the early subacute period (<30 days post-stroke) was performed by Druzbicki et al. (21), but improvements in step symmetry index between the biofeedback and control groups were not statistically significant. However, this study uniquely differed from many other biofeedback RCTs by providing step length biofeedback during bodyweight supported treadmill walking. Overall, there is little research comparing the efficacy of biofeedback interventions for patients in different stages of recovery.

The severity of sensorimotor deficits of stroke participants is another source of variability between studies (Figure 1C). Because there is no strong evidence outlining the clinical characteristics of responders and non-responders to gait biofeedback, inclusion and exclusion criteria tend to differ widely across studies. However, most stroke studies consider cognition, level of gross motor deficit, and level of visual deficit when selecting patients. Exclusion criteria for cognitive deficits have the highest degree of uniformity, with several studies setting a mini-mental state exam score cutoff at 24 for inclusion (21–23). However, other studies fail to include measures of mental acuity in their patient selection process (1). Possibly, a mini-mental cutoff of 24 is conservative for the purposes of generating clean data, and more research should be undertaken to evaluate the relative efficacy of these interventions on individuals with lower mini-mental scores, given that cognitive deficits are common post-stroke (59). Estimates of the prevalence of cognitive deficits following stroke vary, but a large scale study using data from 10 countries found that about 30% of individuals with ischemic stroke had cognitive deficits (mini-mental score <27) (59). Biofeedback interventions may be particularly effective for people whose internal feedback systems are compromised, including those with proprioceptive deficits, as the biofeedback would serve as a substitute for the reduced peripheral input (17). There is also evidence suggesting that perceptual deficits contribute to gait asymmetry in post-stroke individuals, a problem that could be addressed by augmenting sensory input to improve awareness (60). In a study on able-bodied individuals, vibratory stimuli were delivered to targeted muscles (gluteus medius) during gait (stance and swing) to evoke artificial proprioceptive feedback, with promising effects on frontal plane motion during gait (61). However, we could not find studies probing the relationship between sensory impairments post-stroke and response to biofeedback or implementing augmented proprioceptive feedback to retrain post-stroke gait, pointing to another future research area. Overall lower extremity motor deficit is another patient selection criterion, but the measurement tools and cutoff points used to quantify level of deficit vary widely (1, 8, 23). Past studies have included stroke survivors at a specific Brunnstrom stage of recovery (21, 22) or with ability to ambulate a predetermined distance or time (1, 23, 24).

## Summary of Gaps in Previous Research

Due to the varied and often conflicting nature of evidence, more research is needed to prove the efficacy of gait biofeedback for post-stroke individuals (2, 4). However, without a concerted effort to explore the underlying mechanisms that impact the success of gait biofeedback interventions on post-stroke individuals, inconsistent results will likely continue to be observed. Future studies should be designed to directly compare various biofeedback parameters such as feedback target, mode, dosage, etc. in individuals with varied sensorimotor impairments post-stroke.

Our review of previous literature provides several indications of where to begin such future investigations. First, modes of feedback and target parameters could be compared directly across multiple patient populations. Second, motor learning strategies, such as faded feedback or variable practice structure, could be tested against more conventional procedures for the application of biofeedback. Third, greater efforts should be made to identify characteristics of responders and non-responders. Fourth, studies aimed at evaluating dose-response relationships are needed. Fifth, large-sample RCTs are needed, especially for stroke survivors in more acute stages of recovery to compare the relative efficacy of biofeedback for different stages of recovery.

### Neurophysiological and Motor Learning Mechanisms Underlying Gait Biofeedback

As is true for a majority of neuro-rehabilitation treatments, the efficacy of biofeedback as a gait training intervention stands to benefit from a better understanding of the underlying neurophysiological, biomechanical, and motor learning mechanisms of gait biofeedback. There is a research gap related to short-term and long-term changes in cortical and spinal circuitry following gait biofeedback interventions. As an illustration of a study exploring both the biomechanical and neural processes of gait biofeedback, Pietrosimone et al. (62) demonstrated that the use of EMG biofeedback during an isometric force generation task significantly increased both peak knee extension torque and amplitudes of motor evoked potentials elicited from the quadriceps muscles in response to transcranial magnetic stimulation in able bodied individuals (62). The authors argued that these results indicate EMG biofeedback may be a viable strategy for enhancing corticomotor excitability (62). More similar research is needed. The extent to which similar modulation of corticospinal excitability can be achieved during ambulation, with post-stroke individuals, or with other modes of biofeedback remains unknown (63). These gaps in knowledge regarding how and why biofeedback induces its effects on post-stroke gait constitute necessary steps toward developing evidence-based gait biofeedback protocols.

## Perspectives for Future Research Directions Related to Gait Biofeedback

Based on the review of previous research, our long-term vision is that cumulative evidence derived from a series of mechanism-focused and translational gait feedback studies will facilitate the development of clinical prediction rules or decision-making guidelines for more effective and individualized rehabilitation of post-stroke walking function. Completing the studies needed to systematically and individually delineate the multiple variables affecting the success of biofeedback gait training may be time consuming, but the efforts involved would be outweighed by the potential reward of one or more highly effective biofeedback interventions that can later leverage novel wearable sensor technologies.

### Emerging Wearable Sensor Technologies for Gait Biofeedback

Over the past decade, technological evolutions have highlighted novel biofeedback applications to enhance the effectiveness, broaden the application, and advance the clinical impact of gait biofeedback. Wearable sensors can allow a stroke survivor to receive biofeedback *via* portable, non-obtrusive, non-restrictive, user-friendly sensor units on their person, enabling decrease in the cost and increase in the usability of gait biofeedback (64). Many newly developed wearable sensor devices can track gait parameters in real-time, a process which previously required expensive, traditional, laboratory-based biofeedback systems. Recent research efforts have evaluated the validity and reliability of gait outcomes derived from wearable sensors, although more rigorous evaluations of the measurement properties and biofeedback applications of wearable sensors are needed (64, 65). Wearable sensing systems can accurately measure joint angle information during ambulation (17). Kinematic data can be recorded by wearable gyroscopes which measure changes in angular momentum, wearable accelerometers which measure the rate at which a body segment changes speed, and wearable magnetometers capable of capturing a body part's orientation relative to gravity (64). Kinetic variables can be reliably tracked through sensors, such as force sensing in-shoe insoles, or pressure sensing assistive devices (27, 66). Additionally, multiple types of measurement sensors can be combined into a single wearable device to increase overall utility and scope of biofeedback (64). Sensors, such as accelerometers and gyroscopes, have also been used to provide spatiotemporal feedback (67). Similarly, wearable, wireless EMG sensors and EMG biofeedback systems are available (68). Modes of feedback can be user-friendly, and provide cues regarding ongoing gait patterns to the user *via* vibratory or tactile cues emitted from small devices worn by or attached to the patient, or a smartphone (64).

### Promising Clinical Applications of Gait Biofeedback for Home-Based and Tele-Rehabilitation

The accessibility and ease-of-use of new wearable sensor-based gait biofeedback systems may make biofeedback gait training more readily available across diverse practice settings (69, 70). Potentially, a single clinical facility could have a “biofeedback toolbox” with multiple options from which to choose, allowing clinicians to tailor the feedback mode, target, and method that best matches each patient's baseline clinical profile. Wearable sensors can also be compatible with use at home and in community settings (70), enabling high-quality stepping practice outside of the clinic, using customized gait target parameters prescribed by the clinician during in-clinic or tele-rehabilitation evaluations. Considering the established role of repetitive stepping practice in promoting motor recovery, gait biofeedback could become a pivotal addition to a home exercise program for post-stroke individuals (71). The ability to provide skilled, clinician-selected cues without the constant physical presence of a clinician could also have major ramifications for tele-medicine. Cramer et al. (72) found a novel upper extremity telehealth protocol to be non-inferior to in-person therapy for post-stroke individuals, but no lower extremity counterpart exists. Biofeedback can certainly be an important component of tele-rehabilitation treatment protocols, aiding with both retraining of gait quality and remote evaluation of performance, as data gathered through home-based gait biofeedback devices is transmitted from the home to the clinic.

Compared to upper extremity rehabilitation, a tele-rehabilitation system specifically designed for lower extremity or gait biofeedback training would present a series of unique challenges and safety considerations. Ensuring safe implementation would be the foremost concern for both telehealth and conventional home exercise gait biofeedback interventions. Unlike upper limb retraining, gait training requires provision for adequate guarding to prevent falls and injuries during home-exercises. Involvement of the caregiver or supervision from the clinician *via* tele-rehabilitation may help enhance safety and feasibility. Thus, considerable thought and attention will be needed to ensure a suitable environment for gait biofeedback, and to select patients with low fall risk, or with capacity to ambulate short distances without assistance.

Dorsch et al. (73) conducted a large-scale trial in which wearable sensors were used to monitor functional gait variables including walking speed and total walking time in post-stroke individuals, but this study was completed in the inpatient rehabilitation setting, and did not involve the use of real-time biomechanical feedback to improve gait quality. There is a need for more studies assessing the efficacy of wearable sensor-based gait interventions. One systematic review (74) found that incorporation of wearable sensors into gait and balance training had a positive training effect compared to usual care and balance training controls; however, only a few heterogeneous RCTs specifically targeted gait and stroke (74). While the results are promising, more RCTs assessing the efficacy of biofeedback provided *via* wearable sensors especially in community settings are needed.

Finally, there is limited research evaluating the efficacy of gait biofeedback without the presence of a neurorehabilitation clinician or with remote supervision by a clinician. If home-based stepping exercises with gait biofeedback are shown to be safe and at least as effective as home-based walking practice without feedback, gait biofeedback could transform rehabilitation by enhancing patient compliance with home exercise programs, accurately tracking progress and gait performance during community ambulation, and retraining both gait quality and gait speed beyond the clinical therapy sessions. More importantly, biofeedback employed in home-settings may prove to be markedly more effective at promoting neuroplasticity and restoring function than traditional home-exercise programs, due to its ability to enable more precise and higher quality of repetitive stepping practice.

## Conclusions

The current evidence regarding the effectiveness of gait biofeedback following stroke is equivocal, but positive trends indicate promise. Our literature review summarized factors contributing to variability in the results of past studies, such as different types of feedback targets (kinematic, kinetic, spatiotemporal, and EMG), feedback modes (visual, auditory, and haptic), practice structure, dosage, and patient characteristics. Based on our analyses of the gaps in the previous research, we suggest that more favorable and conclusive evidence supporting gait biofeedback can be achieved if larger-sample studies are conducted to directly compare different feedback parameters, employ more uniformity in experimental design, and evaluate patient characteristics of potential responders. The cumulative research evidence gained from an amalgam of mechanism-focused and clinical research will yield clinical prediction rules and decision-making algorithms for optimization of gait biofeedback parameters. Wearable sensor technologies have the potential to transform gait biofeedback and provide greater access and wider array of options for the clinicians while lowering costs. Sensing technologies will be particularly valuable for telehealth and home-based stepping exercise programs. High-quality, portable, user-friendly gait biofeedback systems will provide patients with clinician-determined cues regarding gait performance resulting in a greater number of high-quality repetitions during walking practice. In summary, gait biofeedback has strong potential as a post-stroke gait training tool and warrants further research.

## Author Contributions

JS developed the manuscript draft, conducted the review of literature, and summarized the findings of the review. SW was involved in editing and finalizing the manuscript. TK assisted JS with the literature review, conceptualized the study, and was involved with editing and finalizing the manuscript. All authors contributed to the article and approved the submitted version.

## Conflict of Interest

The authors declare that the research was conducted in the absence of any commercial or financial relationships that could be construed as a potential conflict of interest.
